# New Extension Odd Generalized Rayleigh-Nadarajah Haghighi Distribution with Application and Simulated Data

**DOI:** 10.12688/f1000research.173330.1

**Published:** 2026-02-28

**Authors:** Shihab Ahmed Abdul Reda, Ali Talib Mohammed

**Affiliations:** 1 Department of Mathematics, University of Baghdad College of Education for Pure Science Ibn Al-Haitham, Baghdad, Iraq

**Keywords:** Anderson Darling estimation, Nadarajah Haghighi distributions, T-X family, Cramer Von Mises, quantile function, Order Statistics, Entropy, Simulation

## Abstract

**Background:**

Generating distributions from families is a classification system in the world of probability. Instead of having hundreds of isolated distributions, we groupd them into families based on their shared mathematical properties. This not only organizes our knowledge but also provides powerful tools for analyzing data and building effective statistical models.

**Methods:**

The extended generalized Rayleigh-Nadarajah Haghighi (EOGRNH) distribution was introduced, and its basic statistical features were thoroughly studied. These features include important functions such as the cumulative function (cdf ), probability density function (pdf ), surviva, l and hazard functions. We obtain statistical features including moments, skewness, kurtosis, incomplete moments, order statistics, moment-generating, Rényi entropy and quantile functions. Maximum-Likelihood Estimation (MLE) and Ordinary Least Squares Estimation (OLS) are two common methods for estimating model parameters.

**Results:**

Monte Carlo simulations with different sample sizes (N = 50, 100, 200, and 5000 replications) evaluated the estimator performance using absolute bias and man square error (MSE). When applied to real reliability data, failures of 50 mechanical components per 1000 h, the EOGRNHD outperformed both Gompertz Nadarajah Haghighi (GoNH) and Nadarajah Haghighi (NH) distributions in terms of flexibility and accuracy. The Bayesian information criterion, Anderson-Darling, Hannan information, modified Akaike criterion, Akaike information criterion, Kolmogorov-Smirnov, and Cramer-von-Mises statistics all show that this is especially true under the MLE. The results show that the EOGRNH distribution is useful for reliability analysis and fault modeling.

**Conclusions:**

The EOGRNH was built according to th T-X family of distributions by combining a generator function of extension odd Nadarajah Haghighi with a baseline generalized Rayleigh distribution to ensure the preservation of the properties of the probability. The proposed model can accommodate many known distributions as special cases, thus providing a general mathematical framework for unifying different families of distributions.

## 1. Introduction

Statistical distributions are essential for understanding complex data. Although the T-X family has grown, many traditional distributions are still too rigid to handle different types of data, which makes it difficlt to reflect real-world complexity. In this study, we present an extended generalized Rayleigh-Nadarajah-Haghighi (EGRNH) distribution as a solution. This model uses the single-generalized distribution method within the Rayleigh-Nadarajah-Haghighi framework, giving researchers more flexibility to model varied data and better control over the distribution’s shape and risk. As a result, it is well-suited for a range of practical applications. As a result, various research have investigated the properties and uses of these novel models, including odd Nadarajah Haghighi distribution, ^1^ A novel two-parameter Nadarajah-Haghighi Extension, ^2^ Generalized Exponential Rayleigh Model, ^3^ Exponentiated (Lehmann Type-II) Nadarajah-Haghighi Distribution, ^4^ Nadarajah–Haghighi Lomax, ^5^ New Generalized Nadarajah Haghighi, ^6^ Wavelet-Based Nonparametric Estimation of the Hazard Rate and density Functions: A Simulation Study, ^7, 8^ Simulation-Based Estimation of Two Weibull Distribution Parameters. ^9^ The Gompertz Nadarajah-Haghighi (GoNH) Distribution Properties The Gompertz Nadarajah-Haghigh, ^10^ Our approach extends the method proposed by Alzaatreh, which introduced generating families of continuous probability distributions, ^11^ odd generalized Rayleigh a broad generalization. This makes it a suitable families, ^12^ A Novel Three-Parameter Nadarajah Haghighi. ^13^ The proposed model is used an extension of T-X distributions, especially the EORNH distribution. This model addresses the gaps in previous traditional statistical distributions by offering a greater adaptive potential for modeling data with heavy tails and pronounced skewness. It is significantly superior to the existing distributions in managing complex and long-term data. By offering a powerful and versatile distribution, it accurately and easily maps complex data, especially when standard methods are falter. However previous researches have not found an understandable solution for complex data. Therefore, we propose an extension to the distribution that fills this gap in previous distributions, where we write a new extension to the generalized Rayleigh- Nadarajah-Haghigh distribution. This model is more flexible and is useful for data that do not fit the classical probability models. We used probabilistic methods such as MLE and OLS. We then compared these results with those of the Anderson Darling and Cramervon Mises methods. The results show that the proposed extension provides a more efficient and flexible framework for analyzing non-traditional data in which statistical applications are advanced. In addition, some unconventional data that do not fit the standard statistical assumptions appear here.

This paper proposes a new family, called the extended odd generalized Rayleigh family (EOGR-G). The basis of this family is the generalized Rayleigh distribution. To begin, we define the random variable x > 0 in its odd-generalized form as follows:

$${Ԍ}_{\text{EOGR}-Ԍ}\left(x\right)={\left(1-e^{-b{\left(\frac{{Ƒ}^{a}\left(x;\xi \right)}{1-{Ƒ}^{a}\left(x;\xi \right)}\right)}^{2}}\right)}^{c},x\;\geq \;0;a,b,c>0$$ (1)

$$g_{\text{EOGR}-Ԍ}\left(x\right)=2\mathrm{cb}\left(\frac{\mathrm{af}\left(x;\xi \right)\;{Ƒ}^{2a-1}\left(x;\xi \right)}{{\left(1-{Ƒ}^{a}\left(x;\xi \right)\right)}^{3}}\right)\left(e^{-b{\left(\frac{{Ƒ}^{a}\left(x;\xi \right)}{1-{Ƒ}^{a}\left(x;\xi \right)}\right)}^{2}}\right){\left(1-e^{-b{\left(\frac{{Ƒ}^{a}\left(x;\xi \right)}{1-{Ƒ}^{a}\left(x;\xi \right)}\right)}^{2}}\right)}^{c-1}$$ (2)

## 2. The extension odd Generalized Rayleigh-Nadarajah Haghighi distribution (EOGRNH)

The Nadarajah–Haghighi (NH) distribution is described as a probability distribution with a scale parameter and shape parameter. cdf and pdf are given by

$${Ƒ}_{\mathrm{NH}}\left(x\right)=1-e^{1-{\left(1+kx\right)}^{m}},x>0,m,\delta >0$$ (3)

$$f_{\mathrm{NH}}\left(x\right)=\mathrm{mk}{\left(1+\mathrm{kx}\right)}^{m-1}e^{1-{\left(1+\mathrm{kx}\right)}^{m}}$$ (4)

A new EOGRNHD pdf and cdf are introduced by substituting Equation (3) into Equation (1) and Equation (2) into Equation (4). The results are as follows:

$${Ԍ}_{\text{EOGRNH}}\left(x\right)={\left(1-e^{-b{\left(\frac{{\left(1-e^{1-{\left(1+kx\right)}^{m}}\right)}^{a}}{1-{\left(1-e^{1-{\left(1+kx\right)}^{m}}\right)}^{a}}\right)}^{2}}\right)}^{c}x\;\geq \;0,a,c,b,m,k>0$$ (5)

$$\begin{array}{c} g_{\text{EOGRNH}}\left(x\right)=2\text{acbmk}\;\left(\frac{{\left(1+\mathrm{kx}\right)}^{m-1}e^{1-{\left(1+\mathrm{kx}\right)}^{m}}\;{\left(1-e^{1-{\left(1+\mathrm{kx}\right)}^{m}}\right)}^{2a-1}}{{\left(1-{\left(1-e^{1-{\left(1+\mathrm{kx}\right)}^{m}}\right)}^{a}\right)}^{3}}\right)\\ \left(e^{-b{\left(\frac{{\left(1-e^{1-{\left(1+\mathrm{kx}\right)}^{m}}\right)}^{a}}{1-{\left(1-e^{1-{\left(1+\mathrm{kx}\right)}^{m}}\right)}^{a}}\right)}^{2}}\right){\left(1-e^{-b{\left(\frac{{\left(1-e^{1-{\left(1+\mathrm{kx}\right)}^{m}}\right)}^{a}}{1-{\left(1-e^{1-{\left(1+\mathrm{kx}\right)}^{m}}\right)}^{a}}\right)}^{2}}\right)}^{c-1} \end{array}\;$$ (6)

For the EOGRNHD, we derive the hazard & survival functions by:

$$h_{\text{EOGRNH}}\left(x\right)=\frac{g_{\text{EOGRNH}}\left(x\right)}{1-{Ԍ}_{\text{EOGRNH}}\left(x\right)}\;\text{and}\;S_{\text{EOGRNH}}\left(x\right)=1-{Ԍ}_{\text{EOGRNH}}\left(x\right)$$

$$h_{\text{EOGRNH}}\left(x\right)=\frac{2\text{acbmk}\;\left(\frac{{\left(1+\mathrm{kx}\right)}^{m-1}e^{1-{\left(1+\mathrm{kx}\right)}^{m}}\;{\left(1-e^{1-{\left(1+\mathrm{kx}\right)}^{m}}\right)}^{2a-1}}{{\left(1-{\left(1-e^{1-{\left(1+\mathrm{kx}\right)}^{m}}\right)}^{a}\right)}^{3}}\right)\;\left(e^{-b{\left(\frac{{\left(1-e^{1-{\left(1+\mathrm{kx}\right)}^{m}}\right)}^{a}}{1-{\left(1-e^{1-{\left(1+\mathrm{kx}\right)}^{m}}\right)}^{a}}\right)}^{2}}\right)}{1-{\left(1-e^{-b{\left(\frac{{\left(1-e^{1-{\left(1+\mathrm{kx}\right)}^{m}}\right)}^{a}}{1-{\left(1-e^{1-{\left(1+\mathrm{kx}\right)}^{m}}\right)}^{a}}\right)}^{2}}\right)}^{c}}{\left(1-e^{-b{\left(\frac{{\left(1-e^{1-{\left(1+\mathrm{kx}\right)}^{m}}\right)}^{a}}{1-{\left(1-e^{1-{\left(1+\mathrm{kx}\right)}^{m}}\right)}^{a}}\right)}^{2}}\right)}^{c-1}$$ (7)

$$S_{\text{EOGRNH}}\left(x\right)=1-{\left(1-e^{-b{\left(\frac{{\left(1-e^{1-{\left(1+\mathrm{kx}\right)}^{m}}\right)}^{a}}{1-{\left(1-e^{1-{\left(1+\mathrm{kx}\right)}^{m}}\right)}^{a}}\right)}^{2}}\right)}^{c}$$ (8)

**Figure 1.**
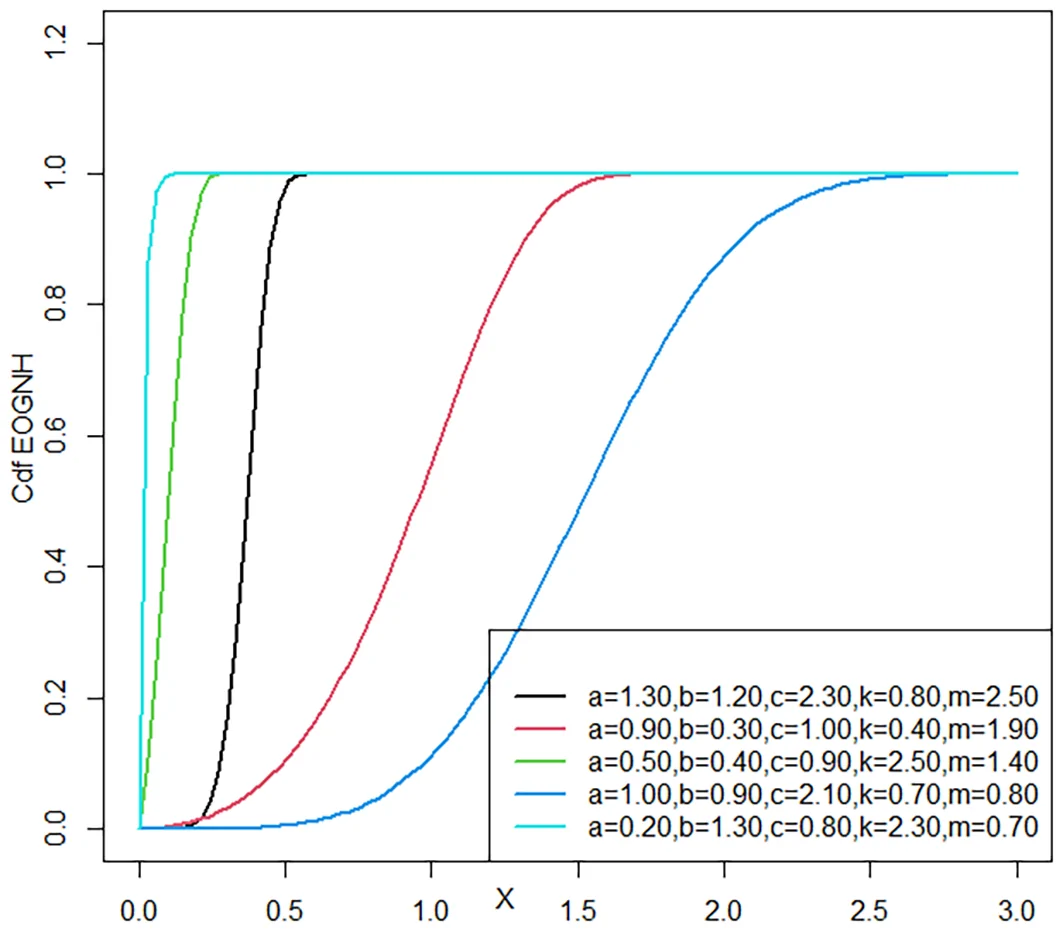
Multiple forms of the (CDF).

Figures 1 and 3 shows the variance of curves representing different distributions and how parameter values affect the shape of the (cdf ). The curves associated with small parameter values show a sharp rise in probabilities at low levels, indicating the density of data around those values. The curves appear at intermediate values to a critical balance point between the rate of increase of the probability and the rate of approach to the threshold value, producing a more stable and balanced distribution. When the parameters are larger, the curves exhibit a more stable behavior, with the rate of increase of probabilities slowing. As the range of possible values increased, the distribution widened. Larger parameter values make the probability curves more balanced because the probability increase more slowly. This means that the distribution covers more values, including higher values, so large outcomes occur more often.

**Figure 2.**
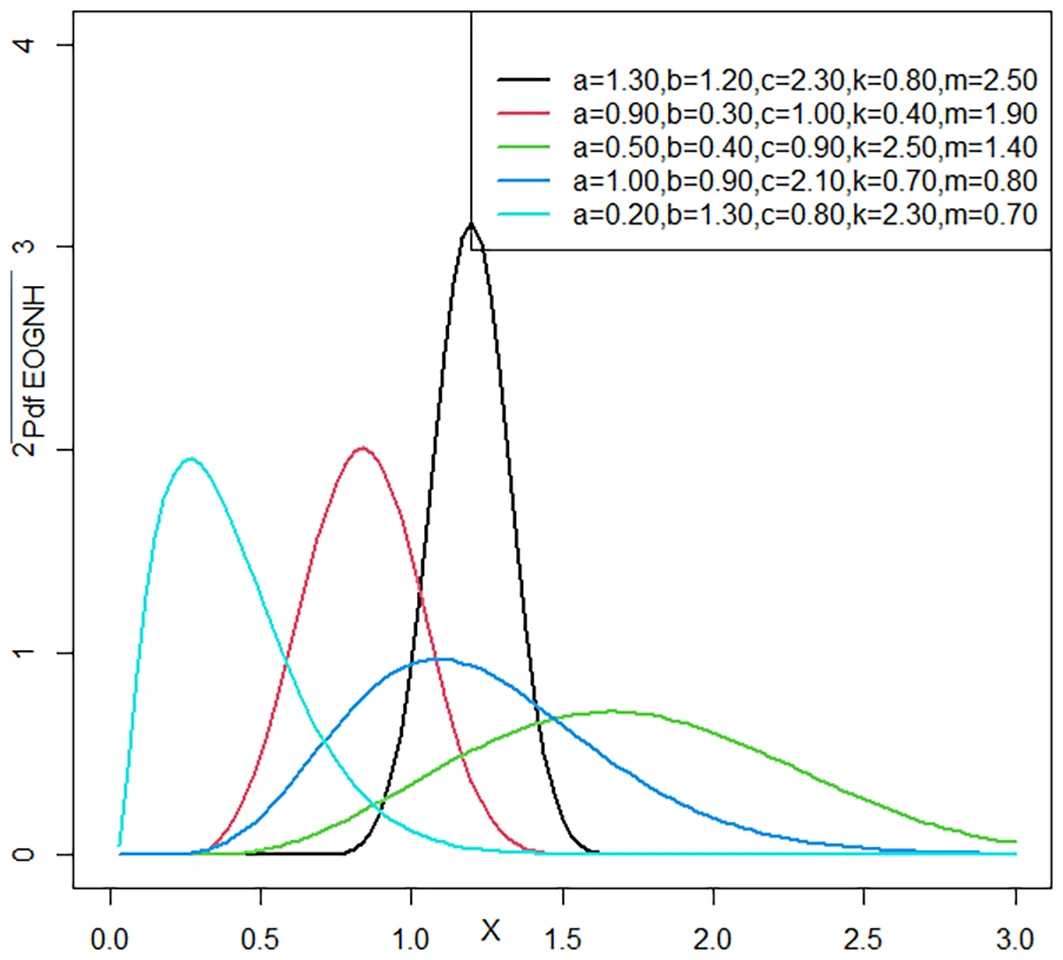
Multiple forms of the (PDF).

**Figure 3.**
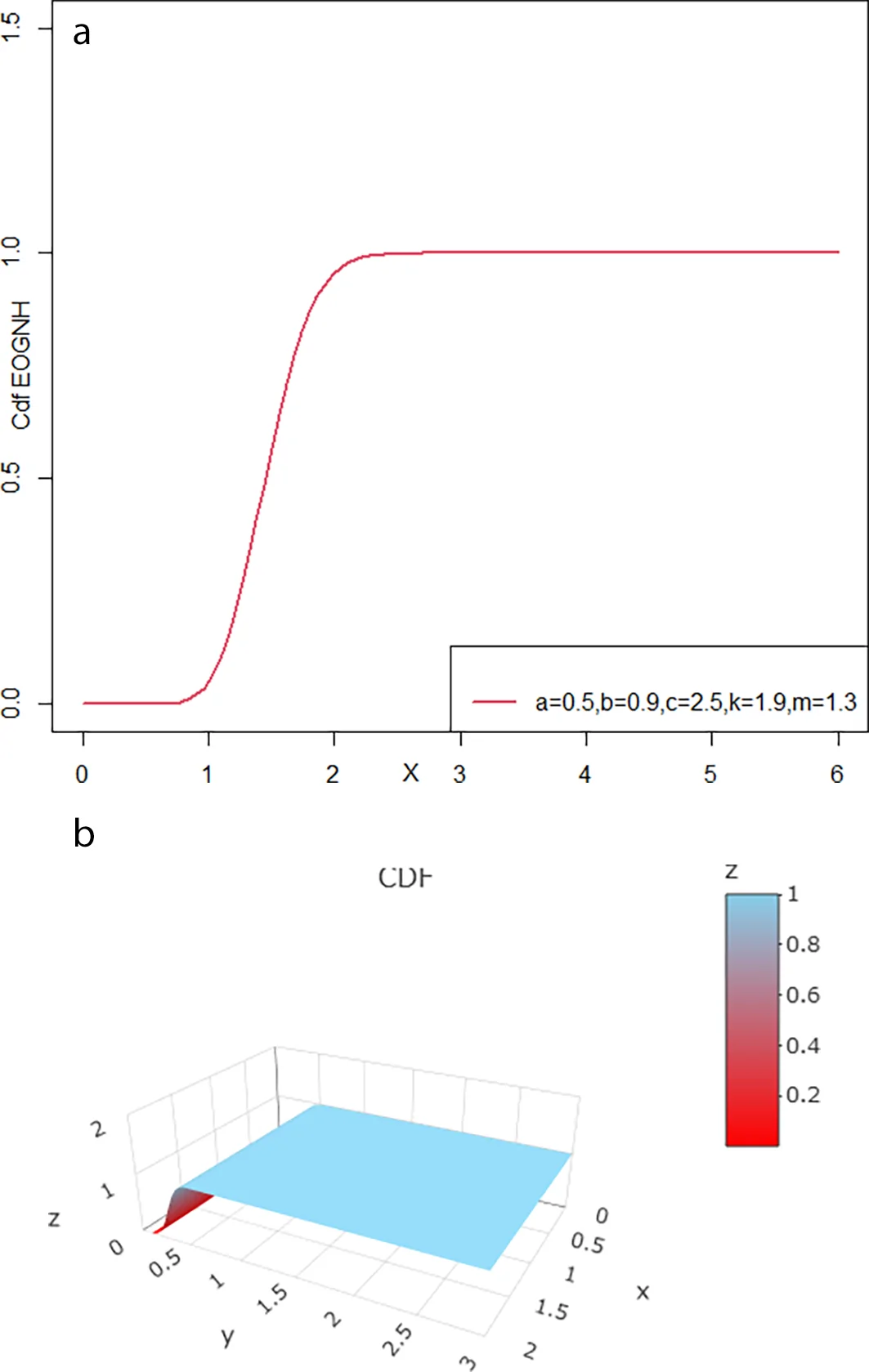
(a) CDF two-dimensional shapes with different parameter values. (b) CDF three-dimensional shapes with different parameter values.

**Figure 4.**
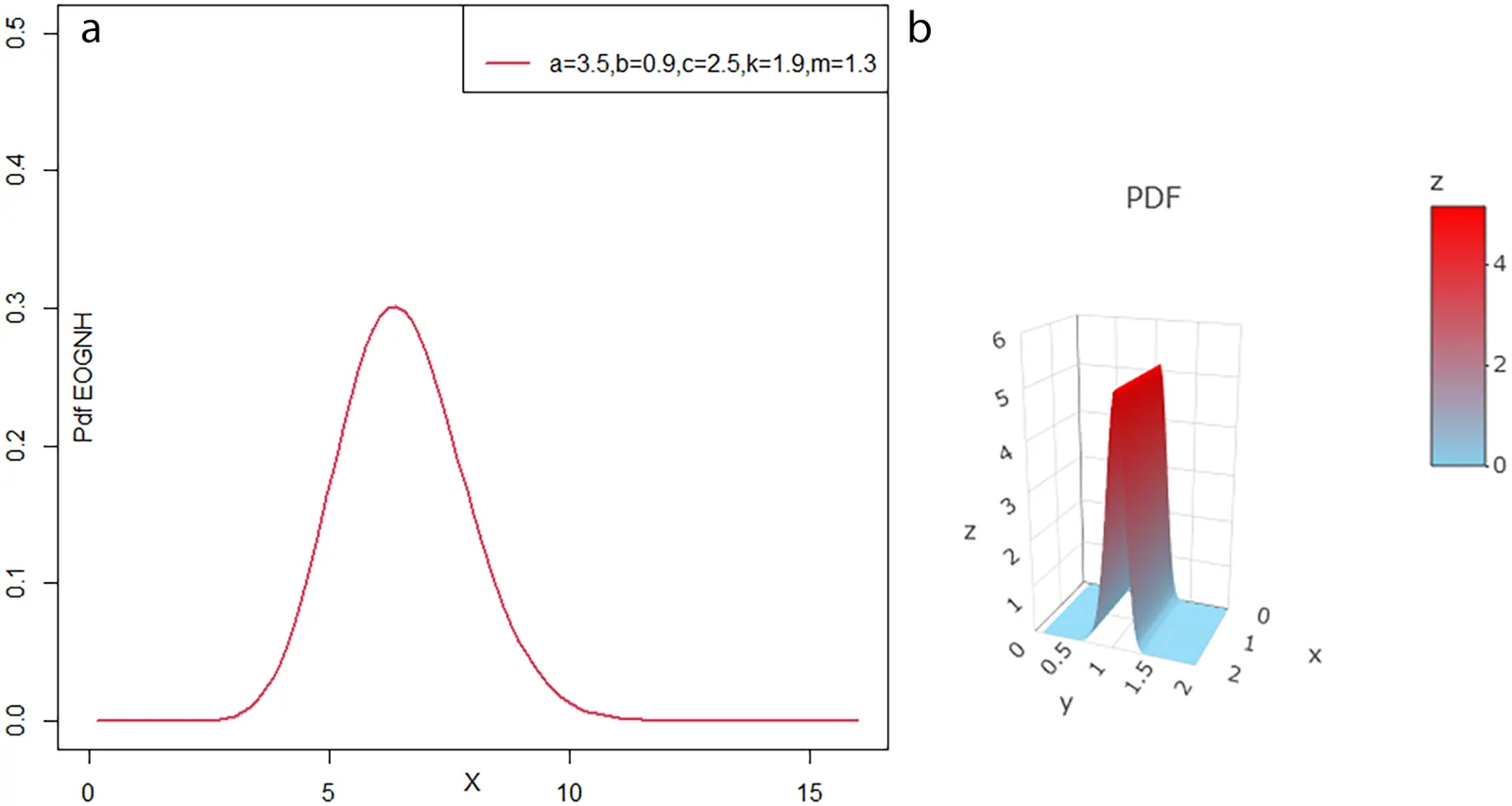
(a) PDF two-dimensional shapes with different parameter values. (b) PDF three-dimensional shapes with different parameter values.

The shapes of the probability density function curves in Figures 2 and 4 depend significantly on the parameter values. When the parameters are small, the curves are sharp and peak, indicating that most data points are closely grouped together. When the parameters are averaged, the curves become flatter and less curved, which means that the data are spread out more evenly, and there is more variation. Therefore, some parameter values may exhibit multi- peak distributions, reflecting the presence of distinct subgroups in the data. Accordingly, the significant impact of changing parameter values on the shape of the PDF curve gives researchers flexibility in choosing the most appropriate models to better analyze and explain the data.

## 3. Methodology and mathematical properties

This section focuses on examining the mathematical properties of the EOGRNH, including the expanded form of the pdf, moments, order statistics, incomplete moments, quantile function, moment-generating function, and Rényi entropy.

### 3.1 Useful expansion

This section presents a formal simplification of Equation (3) by applying the following: $e^{-Z}=\sum_{K=0}^{\infty }\frac{{\left(-Z\right)}^{K}}{K!}$ , [1−Ų]Z=∑i=0∞(Zi)(−Ų)i , ${\left[1-Ų\right]}^{-Z}=\sum_{j=0}^{\infty }\frac{\Gamma \left(Z+j\right)}{j!\Gamma \left(Z\right)}u^{j}$ and |Ų|<1,Z>0. ^14– 16^

By simplifying the equation pdf we get the following equation:

$$g_{\text{EOGR}-G}\left(x\right)=\sum_{i=j=h=0}^{\infty }\left(\begin{array}{c} c-1\\ i \end{array}\right)\frac{{\left(-1\right)}^{i+j}{\left(i+1\right)}^{j}b^{j}\Gamma \left(3+2j+h\right)}{j!h!\Gamma \left(3+2j\right)}2\text{acbf}\left(x\right)\;{Ƒ}^{2a\left(j+1\right)+ah-1}\left(x\right)$$

By applying PDF and CDF Equations (4 & 5) where

$f_{\mathrm{NH}}\left(x\right)=\mathrm{mk}{\left(1+\mathrm{kx}\right)}^{m-1}e^{1-{\left(1+\mathrm{kx}\right)}^{m}}$ and ${Ƒ}^{2a\left(j+1\right)+ah-1}\left(x\right)={\left(1-e^{1-{\left(1+\mathrm{kx}\right)}^{m}}\right)}^{2a\left(j+1\right)+ah-1}$

$$g_{\text{EOGRNH}}\left(x\right)=2\text{acbm}\sum_{i=j=h=v=f=0}^{\infty }\left(\begin{array}{c} m-1\\ f \end{array}\right)\left(\begin{array}{c} c-1\\ i \end{array}\right)\left(\begin{array}{c} 2a\left(j+1\right)+ah-1\\ t \end{array}\right)$$

$$\frac{{\left(-1\right)}^{j+v}\delta^{f+1}{\left(i+1\right)}^{j}b^{j}\Gamma \left(3+2j+h\right)}{j!h!\Gamma \left(3+2j\right)}x^{f}e^{\left(1+v\right)\left(1-{\left(1+\mathrm{kx}\right)}^{m}\right)}$$

$$\mathrm{Let}\;{\text{₭}}_{i,j,h,v,f}=\sum_{i=j=h=v=f=0}^{\infty }\left(\begin{array}{c} 2a\left(j+1\right)+ah-1\\ v \end{array}\right)\left(\begin{array}{c} c-1\\ i \end{array}\right)\left(\begin{array}{c} m-1\\ f \end{array}\right)\frac{{\left(-1\right)}^{j+v}{\left(i+1\right)}^{j}b^{j}\Gamma \left(3+2j+h\right)}{j!h!\Gamma \left(3+2j\right)}$$

$$g{\left(x\right)}_{\text{EOGRNH}}=2{\text{acbm}\text{₭}}_{i,j,h,v,f}\delta^{f+1}x^{f}e^{\left(1+v\right)}e^{-\left(1+v\right){\left(1+\mathrm{kx}\right)}^{m}}$$ (9)

### 3.2 Moment, skewness and kurtosis

It is considered one of the basic properties because, through it, the first and second expectations find variance, skewness and kurtosis ^17^:

$$\mu_{r}^{\prime }=E\left(x^{r}\right)=\int_{0}^{\infty }x^{r}g_{\text{EOGRNH}}\left(x\right)\mathrm{dx}=2{\text{acbm₭}}_{i,j,h,v,f}k^{f+1}e^{\left(1+v\right)}\int_{0}^{\infty }x^{r+f}e^{-\left(1+v\right){\left(1+\mathrm{kx}\right)}^{m}}\mathrm{dx}$$

$$\mathrm{Let}\;y=\left(1+v\right){\left(1+\mathrm{kx}\right)}^{m},\text{then}\;x=\frac{1}{k}\left({\left(\frac{\;y}{\left(1+v\right)}\right)}^{\frac{1}{m}}-1\right),\mathrm{dx}=\frac{1}{\mathrm{mk}\left(1+v\right)}{\left(\frac{\;y}{\left(1+v\right)}\right)}^{\frac{1}{m}-1}\mathrm{dy}$$

$$\text{if}\;\dot{x}=0,\text{then}\;y=1+v\;\text{and if}\;\dot{x}=\infty ,\text{then}\;y=\infty$$

$$\mu_{r}^{\prime }=2\text{acbm}{\text{₭}}_{i,j,h,f,v}k^{f+1}e^{\left(1+v\right)}\int_{1+v}^{\infty }{\left(\frac{1}{k}\left({\left(\frac{\;y}{1+v}\right)}^{\frac{1}{m}}-1\right)\right)}^{r+f}e^{-y}\frac{1}{\mathrm{mk}\left(1+v\right)}{\left(\frac{\;y}{1+v}\right)}^{\frac{1}{m}-1}\mathrm{dy}$$

$${\left({\left(\frac{\;y}{1+v}\right)}^{\frac{1}{m}}-1\right)}^{r+f}=\sum_{s=0}^{\infty }\left(\begin{array}{c} r+f\\ s \end{array}\right){\left(-1\right)}^{s}{\left(\frac{\;y}{1+v}\right)}^{\frac{s}{m}}$$

$$\mu_{r}^{\prime }=2\mathrm{acb}{\text{₭}}_{i,j,h,f,v}\frac{e^{\left(1+v\right)}}{{\left(1+v\right)}^{\frac{s+1}{m}}k^{r}}\;\sum_{s=0}^{\infty }\left(\begin{array}{c} r+f\\ s \end{array}\right){\left(-1\right)}^{s}\int_{1+v}^{\infty }y^{\frac{s+1}{m}-1}e^{-y}\mathrm{dy}$$

$$\mu_{r}^{\prime }={\text{₭}}_{i,j,h,f,v}\frac{2\mathrm{acb}e^{\left(1+v\right)}}{{\left(1+v\right)}^{\frac{s+1}{m}}k^{r}}\;\sum_{s=0}^{\infty }\left(\begin{array}{c} r+f\\ s \end{array}\right){\left(-1\right)}^{s}\;\Gamma \left(\frac{s+1}{m},1+v\right)$$

Let $\left({\text{¥}}_{i,j,h,f,v,s},r\right)={\text{₭}}_{i,j,h,f,v}\frac{2\mathrm{acb}e^{\left(1-v\right)}}{{\left(1-v\right)}^{\frac{s+1}{m}}k^{r}}\;\sum_{s=0}^{\infty }\left(\begin{array}{c} r+f\\ s \end{array}\right){\left(-1\right)}^{s}$

$$\mu_{r}^{\prime }=\left({\text{¥}}_{i,j,h,f,v,s},r\right)\;\Gamma \left(\frac{s+1}{m},1+v\right)$$ (10)

When we use Equation (11) we get:

$$\mu_{1}^{\prime }=\left({\text{¥}}_{i,j,h,f,v,s},1\right)\;\Gamma \left(\frac{s+1}{m},1+v\right)$$

$$\mathrm{var}\left(x\right)=\left({\text{¥}}_{i,j,h,f,v,s},2\right)\;\Gamma \left(\frac{s+1}{m},1+v\right)-{\left(\left({\text{¥}}_{i,j,h,f,v,s},1\right)\;\Gamma \left(\frac{s+1}{m},1+v\right)\right)}^{2}$$

$$\mathrm{SK}=\frac{\left({\text{¥}}_{i,j,h,f,v,s},3\right)\;\Gamma \left(\frac{s+1}{m},1+v\right)}{\sqrt[{2}]{{\left(\left({\text{¥}}_{i,j,h,f,v,s},2\right)\;\Gamma \left(\frac{s+1}{m},1+v\right)\right)}^{3}}}$$

$$\mathrm{SK}=\frac{\left({\text{¥}}_{i,j,h,f,v,s},4\right)\;\Gamma \left(\frac{s+1}{m},1+v\right)}{{\left(\left({\text{¥}}_{i,j,h,f,v,s},2\right)\;\Gamma \left(\frac{s+1}{m},1+v\right)\right)}^{2}}$$

### 3.3 Moment-generating function

It is one of the important properties that we obtain and is given by the following formula:

$${{Ӎ}_{x}}_{\text{EOGRNH}}\left(t\right)=\mu \left(e^{tx}\right)=\int_{0}^{\infty }e^{tx}g_{\text{EOGRNH}}\left(x\right)\mathrm{dx}$$

We’ve substituted Equation (9) ${{Ӎ}_{x}}_{\text{EOGRNH}}\left(t\right)=\mu \left(e^{tx}\right)=2{\text{acbm₭}}_{i,j,h,v,f}k^{f+1}e^{\left(1+v\right)}\int_{0}^{\infty }e^{tx}x^{f}e^{-\left(1+v\right){\left(1+\mathrm{kx}\right)}^{m}}\mathrm{dx}$

$$\mathrm{Let}\;y=\left(1+v\right){\left(1+\mathrm{kx}\right)}^{m},\text{then}\;x=\frac{1}{k}\left({\left(\frac{\;y}{\left(1+v\right)}\right)}^{\frac{1}{m}}-1\right),\mathrm{dx}=\frac{1}{\mathrm{km}\left(1+v\right)}{\left(\frac{\;y}{\left(1+v\right)}\right)}^{\frac{1}{m}-1}\mathrm{dy}$$

$$\text{if}\;\dot{x}=0,\text{then}\;y=1+v\;\text{and if}\;\dot{x}=\infty ,\text{then}\;y=\infty$$

$${{Ӎ}_{x}}_{\text{EOGRNH}}\left(t\right)=2\mathrm{acb}{\text{₭}}_{i,j,h,v,f}e^{\left(1+v\right)}e^{\frac{-t}{k}}\frac{1}{k{\left(1+v\right)}^{1+\frac{1}{m}-1}}\int_{1+v\;}^{\infty }e^{\frac{t}{k}{\left(\frac{\;}{\left(1+v\right)}\right)}^{\frac{1}{m}}}{\left({\left(\frac{\;y}{\left(1+v\right)}\right)}^{\frac{1}{m}}-1\right)}^{f}e^{-y}y^{\frac{1}{m}-1}\mathrm{dy}$$

$$e^{\frac{t}{k}{\left(\frac{\;y}{\left(1+v\right)}\right)}^{\frac{1}{m}}}=\sum_{n=0}^{\infty }\frac{t^{n}}{n!k^{n}}{\left({\left(\frac{\;y}{\left(1+v\right)}\right)}^{\frac{1}{m}}\right)}^{n}$$

$${\left({\left(\frac{\;y}{\left(1+v\right)}\right)}^{\frac{1}{m}}-1\right)}^{f}=\sum_{w=0}^{\infty }\left(\begin{array}{c} f\\ w \end{array}\right){\left(-1\right)}^{w}{\left(\frac{\;y}{\left(1+v\right)}\right)}^{\frac{w}{m}}$$

$${{Ӎ}_{x}}_{\text{EOGRNH}}\left(t\right)=\frac{2\mathrm{acb}{\text{₭}}_{i,j,h,v,f}e^{\left(1+v\right)}e^{\frac{-t}{\delta }}}{\delta {\left(1+v\right)}^{1+\frac{n+1}{\vartheta }+\frac{w}{\vartheta }-1}}\sum_{n=w=0}^{\infty }\left(\begin{array}{c} f\\ w \end{array}\right)\frac{{\left(-1\right)}^{w}t^{n}}{n!\delta^{n}}\int_{1+v\;}^{\infty }y^{\frac{n}{\vartheta }}\;y^{\frac{w}{\vartheta }}e^{-y}y^{\frac{1}{\vartheta }-1}\mathrm{dy}$$

$${{Ӎ}_{x}}_{\text{EOGRNH}}\left(t\right)=\frac{2\mathrm{acb}{\text{₭}}_{i,j,h,v,f}e^{\left(1+v\right)}e^{\frac{-t}{k}}}{k{\left(1+v\right)}^{1+\frac{n+1}{m}+\frac{w}{m}-1}}\sum_{n=w=0}^{\infty }\left(\begin{array}{c} f\\ w \end{array}\right)\frac{{\left(-1\right)}^{w}t^{n}}{n!k^{n}}\int_{1+v\;}^{\infty }y^{\frac{n+w+1}{m}-1}e^{-y}\mathrm{dy}$$

$$\mathrm{Let}\;{Ԋ}_{i,j,h,v,f,n,w}=\frac{2\mathrm{acb}{\text{₭}}_{i,j,h,v,f}e^{\left(1+v\right)}e^{\frac{-t}{k}}}{k{\left(1+v\right)}^{1+\frac{n+1}{m}+\frac{w}{m}-1}}\sum_{n=w=0}^{\infty }\left(\begin{array}{c} f\\ w \end{array}\right)\frac{{\left(-1\right)}^{w}t^{n}}{n!k^{n}}$$

$${{Ӎ}_{x}}_{\text{EOGRNH}}\left(t\right)={Ԋ}_{i,j,h,v,f,n,w}\;\Gamma \left(\frac{n+w+1}{m},1+v\right)$$ (11)

### 3.4 Incomplete moments

Incomplete moments can be obtained from Equation (9) ^18^:

$$\mu_{x}^{\prime }\left(z\right)=\int_{0}^{z}x^{r}\;g\left(x\right)\mathrm{dx}=\int_{0}^{z}x^{r}g\left(x\right)\mathrm{dx}=2{\text{acbm₭}}_{i,j,h,v,f}k^{f+1}e^{\left(1+v\right)}\int_{0}^{z}x^{r+f}e^{-\left(1+v\right){\left(1+\mathrm{kx}\right)}^{m}}\mathrm{dx}$$

$\mathrm{Let}\;y=\left(1+v\right){\left(1+\mathrm{kx}\right)}^{m},\text{then}\;x=\frac{1}{k}\left({\left(\frac{\;y}{\left(1+v\right)}\right)}^{\frac{1}{m}}-1\right),\text{and}$ $\;\mathrm{dx}=\frac{1}{\mathrm{km}\left(1+v\right)}{\left(\frac{\;y}{\left(1+v\right)}\right)}^{\frac{1}{m}-1}\mathrm{dy}$

$$\text{if}\;\dot{x}=0,\text{then}\;y=1+v\;\text{and if}\;\dot{x}=z,\text{then}\;y=\left(1+v\right){\left(1+\mathrm{kz}\right)}^{m}$$

$$\mu_{x}^{\prime }\left(z\right)=2{\text{acbm₭}}_{i,j,h,v,f}k^{-r}e^{\left(1+v\right)}\frac{1}{m{\left(1+v\right)}^{\frac{1}{m}}}\int_{\left(1+v\right)}^{\left(1+v\right){\left(1+\mathrm{kz}\right)}^{m}}{\left({\left(\frac{\;y}{\left(1+v\right)}\right)}^{\frac{1}{m}}-1\right)}^{r+f}e^{-y}y^{\frac{1}{m}-1}\mathrm{dy}$$

$${\left({\left(\frac{\;y}{\left(1+v\right)}\right)}^{\frac{1}{m}}-1\right)}^{r+f}=\sum_{s=0}^{\infty }\left(\begin{array}{c} r+f\\ s \end{array}\right){\left(-1\right)}^{s}{\left(\frac{\;y}{\left(1+v\right)}\right)}^{\frac{s}{m}}$$

$$\mu_{x}^{\prime }\left(z\right)=2{\text{acbm₭}}_{i,j,h,v,f}k^{-r}e^{\left(1+v\right)}\frac{1}{m{\left(1+v\right)}^{\frac{1+s}{m}}}\;\sum_{s=0}^{\infty }\left(\begin{array}{c} r+f\\ s \end{array}\right){\left(-1\right)}^{s}\int_{\left(1+v\right)}^{\left(1+v\right){\left(1+\mathrm{kz}\right)}^{m}}y^{\frac{s+1}{m}-1}e^{-y}\mathrm{dy}$$

$$\left({\text{¥}}_{i,j,h,f,v,s},r\right)=2{\text{acbm₭}}_{i,j,h,v,f}k^{-r}e^{\left(1+v\right)}\frac{1}{m{\left(1+v\right)}^{\frac{1+s}{m}}}\;\sum_{s=0}^{\infty }\left(\begin{array}{c} r+f\\ s \end{array}\right){\left(-1\right)}^{s}$$

$$\mu_{x}^{\prime }\left(z\right)={\text{¥}}_{i,j,h,f,v,s}\;\left(\Gamma \left(\frac{s+1}{v},\left(1+v\right){\left(1+\mathrm{kz}\right)}^{m}\right)-\Gamma \left(\frac{s+1}{v},1+v\right)\right)$$ (12)

### 3.5 Quantile function

The quantile function is produced by inverse Equation (6):

$$U={\left(1-e^{-b{\left(\frac{{\left(1-e^{1-{\left(1+\mathrm{kx}\right)}^{m}}\right)}^{a}}{1-{\left(1-e^{1-{\left(1+\mathrm{kx}\right)}^{m}}\right)}^{a}}\right)}^{2}}\right)}^{c}$$

$$G^{-1}\left(U\right)=x_{U}=\frac{1}{k}\left({\left(1-\ln\left(1-{\left(\frac{\sqrt{\frac{-1}{b}\ln\left(1-U^{\frac{1}{c}}\right)}}{1+\sqrt{\frac{-1}{b}\ln\left(1-U^{\frac{1}{c}}\right)}}\right)}^{\frac{1}{a}}\right)\right)}^{\frac{1}{m}}-1\right)$$ (13)

### 3.6 Order statistic

Let $x_{1:N}\;\leq \;x_{2:N}\;\leq \;x_{3:N}\;\leq \;\ldots \;\leq \;x_{N:N}$ be the order statistics of size K from EOGRNH. pdf is defined as follows. ^19^

$$g_{P,N}\left(x\right)=\frac{N!}{\left(P-1\right)!\left(N-P\right)!}{\left(G_{\text{EOGRNH}}\left(x\right)\right)}^{P-1}{\left(1-G_{\text{EOGRNH}}\left(x\right)\right)}^{N-P}g_{\text{EOGRNH}}\left(x\right)$$

From Equations (5 & 6) into $g_{p,N}\left(x\right)$ , and the result is as follows:

$$\begin{array}{c} g_{P,N}\left(x\right)=\frac{N!}{\left(P-1\right)!\left(N-P\right)!}2\text{acbmk}{\left[1-{\left(1-e^{-b{\left(\frac{{\left(1-e^{1-{\left(1+\mathrm{kx}\right)}^{m}}\right)}^{a}}{1-{\left(1-e^{1-{\left(1+\mathrm{kx}\right)}^{m}}\right)}^{a}}\right)}^{2}}\right)}^{c}\right]}^{N-P}{\left(1-e^{-b{\left(\frac{{\left(1-e^{1-{\left(1+\mathrm{kx}\right)}^{m}}\right)}^{a}}{1-{\left(1-e^{1-{\left(1+\mathrm{kx}\right)}^{m}}\right)}^{a}}\right)}^{2}}\right)}^{cP-1}\\ \left(\frac{{\left(1+\mathrm{kx}\right)}^{m-1}e^{1-{\left(1+\mathrm{kx}\right)}^{m}}\;{\left(1-e^{1-{\left(1+\mathrm{kx}\right)}^{\vartheta }}\right)}^{2a-1}}{{\left(1-{\left(1-e^{1-{\left(1+\mathrm{kx}\right)}^{m}}\right)}^{a}\right)}^{3}}\right)\;e^{-b{\left(\frac{{\left(1-e^{1-{\left(1+\mathrm{kx}\right)}^{m}}\right)}^{a}}{1-{\left(1-e^{1-{\left(1+\mathrm{kx}\right)}^{m}}\right)}^{a}}\right)}^{2}} \end{array}$$ (14)

### 3.7 Rėnyi entropy

One of the important characteristics that play a fundamental role in extracting information is the measure of randomness defined as follows:

$${Ŧ}_{R}\left(Ŋ\right)=\frac{1}{1-Ŋ}\log\int_{0}^{\infty }{g^{Ŋ}}_{\text{EOGNH}}\left(x\right)\mathrm{dx}$$

By using Equation (6), we get:

ŦR(Ŋ)=11−Ŋlog∫0∞[2acbkm((1+kx)m−1e1−(1+kx)m(1−e1−(1+kx)m)2a−1(1−(1−e1−(1+kx)m)a)3)(e−b((1−e1−(1+kx)m)a1−(1−e1−(1+kx)m)a)2)(1−e−b((1−e1−(1+kx)m)a1−(1−e1−(1+kx)m)a)2)c−1]Ŋdx (15)

## 4. Estimation methods

### 4.1 Maximum likelihood estimation

Let $x_{1}x_{2},\ldots ..,x_{N}$ be a randomsample of EOGRNH. The likelihood is:

$$\underset{-}{L}\left(x;a,b,c,k,m\right)=\prod_{i=1}^{N}g\left(x;a,b,c,k,m\right)$$

$$\underset{-}{L}\left(x;a,b,c,k,m\right)=\prod_{i=1}^{N}2\text{acbkm}\;\left(\frac{{\left(1+kx_{i}\right)}^{m-1}e^{1-{\left(1+kx_{i}\right)}^{m}}\;{\left(1-e^{1-{\left(1+kx_{i}\right)}^{m}}\right)}^{2a-1}}{{\left(1-{\left(1-e^{1-{\left(1+kx_{i}\right)}^{m}}\right)}^{a}\right)}^{3}}\right)\;\left(e^{-b{\left(\frac{{\left(1-e^{1-{\left(1+kx_{i}\right)}^{m}}\right)}^{a}}{1-{\left(1-e^{1-{\left(1+kx_{i}\right)}^{m}}\right)}^{a}}\right)}^{2}}\right){\left(1-e^{-b{\left(\frac{{\left(1-e^{1-{\left(1+kx_{i}\right)}^{m}}\right)}^{a}}{1-{\left(1-e^{1-{\left(1+kx_{i}\right)}^{m}}\right)}^{a}}\right)}^{2}}\right)}^{c-1}$$

$$\underset{-}{L}\left(x;a,b,c,k,m\right)=2^{N}a^{N}c^{N}b^{N}m^{N}k^{N}\prod_{i=1}^{N}\left(\frac{{\left(1+kx_{i}\right)}^{m-1}e^{1-{\left(1+kx_{i}\right)}^{m}}\;{\left(1-e^{1-{\left(1+kx_{i}\right)}^{m}}\right)}^{2a-1}}{{\left(1-{\left(1-e^{1-{\left(1+kx_{i}\right)}^{m}}\right)}^{a}\right)}^{3}}\right)\;\left(e^{-b{\left(\frac{{\left(1-e^{1-{\left(1+kx_{i}\right)}^{m}}\right)}^{a}}{1-{\left(1-e^{1-{\left(1+kx_{i}\right)}^{m}}\right)}^{a}}\right)}^{2}}\right){\left(1-e^{-b{\left(\frac{{\left(1-e^{1-{\left(1+kx_{i}\right)}^{m}}\right)}^{a}}{1-{\left(1-e^{1-{\left(1+kx_{i}\right)}^{m}}\right)}^{a}}\right)}^{2}}\right)}^{c-1}$$

The log likelihood of EOGRNH:

$$\ell =N\log2+N\text{loga}+N\text{logc}+N\text{logb}+N\text{logm}+N\text{logk}+\left(m-1\right)\sum_{i=1}^{N}\log\left(1+kx_{i}\right)+\sum_{i=1}^{N}\left(1-{\left(1+kx_{i}\right)}^{m}\right)+\left(2a-1\right)\sum_{i=1}^{N}\left(1-e^{1-{\left(1+kx_{i}\right)}^{m}}\right)-3\sum_{i=1}^{N}\log\left(1-{\left(1-e^{1-{\left(1+kx_{i}\right)}^{m}}\right)}^{a}\right)-b\sum_{i=1}^{N}{\left(\frac{{\left(1-e^{1-{\left(1+kx_{i}\right)}^{m}}\right)}^{a}}{1-{\left(1-e^{1-{\left(1+kx_{i}\right)}^{m}}\right)}^{a}}\right)}^{2}+\left(c-1\right)\sum_{i=1}^{N}\log\left(1-e^{-b{\left(\frac{{\left(1-e^{1-{\left(1+kx_{i}\right)}^{m}}\right)}^{a}}{1-{\left(1-e^{1-{\left(1+kx_{i}\right)}^{m}}\right)}^{a}}\right)}^{2}}\right)$$ (16)

To estimate each parameter, we calculated the derivatives of the logarithm from parameters a,b,c,kandm , as follows:

$$\frac{\partial \left(\ell \right)}{\partial c}=\frac{N}{c}+\sum_{i=1}^{N}\log\left(1-e^{-b{\left(\frac{{\left(1-e^{1-{\left(1+kx_{i}\right)}^{m}}\right)}^{a}}{1-{\left(1-e^{1-{\left(1+kx_{i}\right)}^{m}}\right)}^{a}}\right)}^{2}}\right)$$ (17)

$$\frac{\partial \left(\ell \right)}{\partial b}=\frac{N}{b}-\sum_{i=1}^{N}{\left(\frac{{\left(1-e^{1-{\left(1+kx_{i}\right)}^{m}}\right)}^{a}}{1-{\left(1-e^{1-{\left(1+kx_{i}\right)}^{m}}\right)}^{a}}\right)}^{2}+\left(c-1\right)\sum_{i=1}^{N}\frac{{\left(\frac{{\left(1-e^{1-{\left(1+kx_{i}\right)}^{m}}\right)}^{a}}{1-{\left(1-e^{1-{\left(1+kx_{i}\right)}^{m}}\right)}^{a}}\right)}^{2}e^{-b{\left(\frac{{\left(1-e^{1-{\left(1+kx_{i}\right)}^{m}}\right)}^{a}}{1-{\left(1-e^{1-{\left(1+kx_{i}\right)}^{m}}\right)}^{a}}\right)}^{2}}}{1-e^{-b{\left(\frac{{\left(1-e^{1-{\left(1+kx_{i}\right)}^{m}}\right)}^{a}}{1-{\left(1-e^{1-{\left(1+kx_{i}\right)}^{m}}\right)}^{a}}\right)}^{2}}}$$ (18)

$$\frac{\partial \left(\ell \right)}{\partial k}=\frac{N}{k}\left(m-1\right)\sum_{i=1}^{N}\frac{x_{i}}{1-kx_{i}}-m\sum_{i=1}^{N}x_{i}{\left(1-kx_{i}\right)}^{m-1}-\left(2a-1\right)m\sum_{i=1}^{N}x_{i}e^{1-{\left(1+kx_{i}\right)}^{m}}{\left(1+kx_{i}\right)}^{m-1}+3\mathrm{am}\sum_{i=1}^{N}\frac{{\left(1-e^{1-{\left(1+kx_{i}\right)}^{m}}\right)}^{a-1}}{1-{\left(1-e^{1-{\left(1+kx_{i}\right)}^{m}}\right)}^{a}}e^{1-{\left(1+kx_{i}\right)}^{m}}{\left(1+kx_{i}\right)}^{m-1}x_{i}-b\sum_{i=1}^{N}\frac{2\mathrm{am}{\left(1-e^{1-{\left(1+kx_{i}\right)}^{m}}\right)}^{2a-1}e^{1-{\left(1+kx_{i}\right)}^{m}}{\left(1+kx_{i}\right)}^{m-1}x}{{\left(1-{\left(1-e^{1-{\left(1+kx_{i}\right)}^{m}}\right)}^{a}\right)}^{3}}+2\mathrm{abm}\left(c-1\right)\sum_{i=1}^{N}\frac{e^{-b{\left(\frac{{\left(1-e^{1-{\left(1+kx_{i}\right)}^{m}}\right)}^{a}}{1-{\left(1-e^{1-{\left(1+kx_{i}\right)}^{m}}\right)}^{a}}\right)}^{2}}{\left(1+kx_{i}\right)}^{m-1}{\left(1-e^{1-{\left(1+kx_{i}\right)}^{m}}\right)}^{a-1}e^{-{\left(1+kx_{i}\right)}^{m}}x_{i}}{\left(1-e^{-b{\left(\frac{{\left(1-e^{1-{\left(1+kx_{i}\right)}^{m}}\right)}^{a}}{1-{\left(1-e^{1-{\left(1+kx_{i}\right)}^{m}}\right)}^{a}}\right)}^{2}}\right){\left(1-{\left(1-e^{1-{\left(1+kx_{i}\right)}^{m}}\right)}^{a}\right)}^{2}}$$ (19)

$$\begin{array}{c} \frac{\partial \left(\ell \right)}{\partial a}=\frac{N}{a}+2\sum_{i=0}^{N}\left(1-e^{1-{\left(1+kx_{i}\right)}^{m}}\right)+\sum_{i=1}^{N}\frac{{\left(1-e^{1-{\left(1+kx_{i}\right)}^{m}}\right)}^{2a}\ln\left(1-e^{1-{\left(1+kx_{i}\right)}^{m}}\right)}{{\left(1-{\left(1-e^{1-{\left(1+kx_{i}\right)}^{m}}\right)}^{a}\right)}^{3}}\\ \left(-2b+2\left(c-1\right)b\left(\frac{e^{-b{\left(\frac{{\left(1-e^{1-{\left(1+kx_{i}\right)}^{m}}\right)}^{a}}{1-{\left(1-e^{1-{\left(1+kx_{i}\right)}^{m}}\right)}^{a}}\right)}^{2}}}{\left(1-e^{-b{\left(\frac{{\left(1-e^{1-{\left(1+kx_{i}\right)}^{m}}\right)}^{a}}{1-{\left(1-e^{1-{\left(1+\mathrm{\delta k}\right)}^{m}}\right)}^{a}}\right)}^{2}}\right)}\right)\right) \end{array}$$ (20)

$$\begin{array}{c} \frac{\partial \left(\ell \right)}{\partial m}=\frac{N}{m}+m\sum_{i=1}^{N}\log\left(1+kx_{i}\right)-\sum_{i=1}^{N}{\left(1+kx_{i}\right)}^{m}\ln\left(1+kx_{i}\right)-\left(2a-1\right)\\ \sum_{i=1}^{N}e^{1-{\left(1+kx_{i}\right)}^{m}}\ln\left(1+kx_{i}\right){\left(1+kx_{i}\right)}^{m}-3\sum_{i=1}^{N}\frac{a{\left(1-e^{1-{\left(1+kx_{i}\right)}^{m}}\right)}^{a-1}}{{\left(1-e^{1-{\left(1+kx_{i}\right)}^{m}}\right)}^{a}}\\ \begin{array}{c} e^{1-{\left(1+kx_{i}\right)}^{m}}\ln\left(1+kx_{i}\right){\left(1+kx_{i}\right)}^{m}\\ -2ab\sum_{i=1}^{N}\left(\frac{{\left(1-e^{1-{\left(1+kx_{i}\right)}^{m}}\right)}^{a}}{1-{\left(1-e^{1-{\left(1+kx_{i}\right)}^{m}}\right)}^{a}}\right)\frac{{\left(1-e^{1-{\left(1+kx_{i}\right)}^{m}}\right)}^{a-1}e^{1-{\left(1+kx_{i}\right)}^{m}}{\left(1+kx_{i}\right)}^{m}}{{\left(1-{\left(1-e^{1-{\left(1+kx_{i}\right)}^{m}}\right)}^{a}\right)}^{2}}\\ \begin{array}{c} \ln\left(1+kx_{i}\right)+\left(c-1\right)2\mathrm{ab}\;e^{-b\left(\frac{{\left(1-e^{1-{\left(1+kx_{i}\right)}^{m}}\right)}^{a}}{1-{\left(1-e^{1-{\left(1+kx_{i}\right)}^{m}}\right)}^{a}}\right)}\\ \frac{{\left(1-e^{1-{\left(1+kx_{i}\right)}^{m}}\right)}^{2a-1}e^{1-{\left(1+kx_{i}\right)}^{m}}{\left(1+kx_{i}\right)}^{m}\ln\left(1+kx_{i}\right)}{{\left(1-{\left(1-e^{1-{\left(1+kx_{i}\right)}^{m}}\right)}^{a}\right)}^{3}} \end{array} \end{array} \end{array}$$ (21)

The above equations are set equal to zero and subsequently solved using R to obtain the estimated values of the parameters.

### 4.2 Ordinary least squares estimation method

OLS is the most popular technique for estimating the linear or nonlinear variables that constitute the model parameters. The primary goal of this method is to minimize the sum of squared differences between the predicted values and the observed sample values regressed by the OLS. ^20, 21^

$$\text{₩}\left(x;a,b,c,k,m\right)=\sum_{i=0}^{n}{ᶓ}_{i}^{2}=\sum_{i=0}^{n}{\left({Ԍ}_{\text{EOGRNH}}\left(x\right)-\frac{i}{n+1}\right)}^{2}=\sum_{i=0}^{n}{\left({\left(1-e^{-b{\left(\frac{{\left(1-e^{1-{\left(1+kx_{i}\right)}^{m}}\right)}^{a}}{1-{\left(1-e^{1-{\left(1+kx_{i}\right)}^{m}}\right)}^{a}}\right)}^{2}}\right)}^{c}-\frac{i}{n+1}\right)}^{2}$$

$$\begin{array}{c} \frac{\partial \text{₩}}{\partial a}=4\mathrm{cb}\sum_{i=0}^{n}\left({\left(1-e^{-b{\left(\frac{{\left(1-e^{1-{\left(1+kx_{i}\right)}^{m}}\right)}^{a}}{1-{\left(1-e^{1-{\left(1+kx_{i}\right)}^{m}}\right)}^{a}}\right)}^{2}}\right)}^{c}-\frac{i}{n+1}\right)\\ {\left(1-e^{-b{\left(\frac{{\left(1-e^{1-{\left(1+kx_{i}\right)}^{m}}\right)}^{a}}{1-{\left(1-e^{1-{\left(1+kx_{i}\right)}^{m}}\right)}^{a}}\right)}^{2}}\right)}^{c-1}e^{-b{\left(\frac{{\left(1-e^{1-{\left(1+kx_{i}\right)}^{m}}\right)}^{a}}{1-{\left(1-e^{1-{\left(1+kx_{i}\right)}^{m}}\right)}^{a}}\right)}^{2}}\\ \frac{{\left(1-e^{1-{\left(1+kx_{i}\right)}^{m}}\right)}^{2a}\ln\left(1-e^{1-{\left(1+kx_{i}\right)}^{a}}\right)}{{\left(1-{\left(1-e^{1-{\left(1+kx_{i}\right)}^{m}}\right)}^{a}\right)}^{3}} \end{array}$$ (22)

$$\begin{array}{c} \frac{\partial \text{₩}}{\partial m}=2\mathrm{acb}\sum_{i=0}^{n}1-e^{-b{\left(\frac{{\left(1-e^{1-{\left(1+kx_{i}\right)}^{m}}\right)}^{a}}{1-{\left(1-e^{1-{\left(1+kx_{i}\right)}^{m}}\right)}^{a}}\right)}^{2}}{\left(1-e^{-b{\left(\frac{{\left(1-e^{1-{\left(1+kx_{i}\right)}^{m}}\right)}^{a}}{1-{\left(1-e^{1-{\left(1+kx_{i}\right)}^{m}}\right)}^{a}}\right)}^{2}}\right)}^{c-1}\\ e^{-b{\left(\frac{{\left(1-e^{1-{\left(1+kx_{i}\right)}^{m}}\right)}^{a}}{1-{\left(1-e^{1-{\left(1+kx_{i}\right)}^{m}}\right)}^{a}}\right)}^{2}}\frac{{\left(1-e^{1-{\left(1+kx_{i}\right)}^{m}}\right)}^{a}}{1-{\left(1-e^{1-{\left(1+kx_{i}\right)}^{m}}\right)}^{a}}\\ \left(\frac{{\left(1-e^{1-{\left(1+kx_{i}\right)}^{m}}\right)}^{a-1}e^{1-{\left(1+kx_{i}\right)}^{m}}\ln\left(1+kx_{i}\right){\left(1+kx_{i}\right)}^{m}}{{\left(1-{\left(1-e^{1-{\left(1+kx_{i}\right)}^{m}}\right)}^{a}\right)}^{2}}\right) \end{array}$$ (23)

$$\begin{array}{c} \frac{\partial \text{₩}}{\partial k}=4\text{abcm}\sum_{i=0}^{n}{\left({\left(1-e^{-b{\left(\frac{{\left(1-e^{1-{\left(1+kx_{i}\right)}^{m}}\right)}^{a}}{1-{\left(1-e^{1-{\left(1+kx_{i}\right)}^{m}}\right)}^{a}}\right)}^{2}}\right)}^{c}-\frac{i}{n+1}\right)}^{2}{\left(1-e^{-b{\left(\frac{{\left(1-e^{1-{\left(1+kx_{i}\right)}^{m}}\right)}^{a}}{1-{\left(1-e^{1-{\left(1+kx_{i}\right)}^{m}}\right)}^{a}}\right)}^{2}}\right)}^{c-1}\frac{{\left(1-e^{1-{\left(1+kx_{i}\right)}^{m}}\right)}^{a}}{1-{\left(1-e^{1-{\left(1+kx_{i}\right)}^{\vartheta }}\right)}^{a}}\\ e^{-b{\left(\frac{{\left(1-e^{1-{\left(1+kx_{i}\right)}^{m}}\right)}^{a}}{1-{\left(1-e^{1-{\left(1+kx_{i}\right)}^{m}}\right)}^{a}}\right)}^{2}}\frac{{\left(1-e^{1-{\left(1+kx_{i}\right)}^{m}}\right)}^{a-1}e^{1-{\left(1+kx_{i}\right)}^{m}}x_{i}{\left(1+kx_{i}\right)}^{m-1}}{{\left(1-{\left(1-e^{1-{\left(1+kx_{i}\right)}^{m}}\right)}^{a}\right)}^{2}} \end{array}$$ (24)

$$\begin{array}{c} \frac{\partial \text{₩}}{\partial b}=2c\sum_{i=0}^{n}\left({\left(1-e^{-b{\left(\frac{{\left(1-e^{1-{\left(1+kx_{i}\right)}^{m}}\right)}^{a}}{1-{\left(1-e^{1-{\left(1+kx_{i}\right)}^{m}}\right)}^{a}}\right)}^{2}}\right)}^{c}-\frac{i}{n+1}\right){\left(1-e^{-b{\left(\frac{{\left(1-e^{1-{\left(1+kx_{i}\right)}^{m}}\right)}^{a}}{1-{\left(1-e^{1-{\left(1+kx_{i}\right)}^{m}}\right)}^{a}}\right)}^{2}}\right)}^{c-1}\\ {\left(\frac{{\left(1-e^{1-{\left(1+kx_{i}\right)}^{m}}\right)}^{a}}{1-{\left(1-e^{1-{\left(1+kx_{i}\right)}^{m}}\right)}^{a}}\right)}^{2}e^{-b{\left(\frac{{\left(1-e^{1-{\left(1+kx_{i}\right)}^{m}}\right)}^{a}}{1-{\left(1-e^{1-{\left(1+kx_{i}\right)}^{m}}\right)}^{a}}\right)}^{2}} \end{array}$$ (25)

$$\frac{\partial \text{₩}}{\partial c}=2\sum_{i=0}^{n}{\left({\left(1-e^{-b{\left(\frac{{\left(1-e^{1-{\left(1+kx_{i}\right)}^{m}}\right)}^{a}}{1-{\left(1-e^{1-{\left(1+kx_{i}\right)}^{m}}\right)}^{a}}\right)}^{2}}\right)}^{c}-\frac{i}{n+1}\right)}^{2}{\left(1-e^{-b{\left(\frac{{\left(1-e^{1-{\left(1+kx_{i}\right)}^{m}}\right)}^{a}}{1-{\left(1-e^{1-{\left(1+kx_{i}\right)}^{m}}\right)}^{a}}\right)}^{2}}\right)}^{c}\ln\left(1-e^{-b{\left(\frac{{\left(1-e^{1-{\left(1+kx_{i}\right)}^{m}}\right)}^{a}}{1-{\left(1-e^{1-{\left(1+kx_{i}\right)}^{m}}\right)}^{a}}\right)}^{2}}\right)$$ (26)

## 5. Simulation

Maximum-likelihood and ordinary-least-squares methods were used to estimate the EOGRNH distribution parameters. The simulation study is compared by evaluating the average values of the three measured quantities.: absolute Bais $\left|\text{Baisτ}|=\frac{1}{N}\sum_{i=1}^{N}|\hat{\tau }-\tau \right|$ *,* mean square error (MSE), $\mathrm{MSE}=\frac{1}{N}\sum_{i=1}^{N}{\left(\hat{\tau }-\tau \right)}^{2}.$ To perform the simulation, observations from the EOGRNH distribution were generated using U as a uniform r.v. defined in [0,1]. We generate N = 5,000 random samples, and sample sizes *n = 5, 100* and *200* from the EOGRNH with two different sets of initial parameter values. For each parameter combination and sample, we estimated the EOGRNH parameters, a,b,c,k and m using two different estimators which are OLS and MLE. Subsequently, |Baisτ| and MSEs of the parameter estimates were computed. Simulated outcomes are shown in Tables 1 & 2.

**Table 1.** The results of simulations conducted for the EOGRNH distribution are reported for τ *=* ( *a = 1.3, b = 1.03*, c = 1.11, k=0.5,m=3.9 ) *^T^.*

n	Est.	Est. Par.	MLE	OLSE
50	**MSE**	$\hat{a}$	1.7524	0.3529
		$\hat{b}$	1.6362	5.5785
		$\hat{c}$	1.8306	1.0418
		$\hat{k}$	0.0398	0.0115
		$\hat{m}$	2.4673	1.0086
	**|BIAS|**	$\hat{a}$	0.0121	0.2037
		$\hat{b}$	0.3657	1.4018
		$\hat{c}$	0.5897	0.7474
		$\hat{k}$	0.0575	0.0560
		$\hat{m}$	0.0098	0.3965
100	**MSE**	$\hat{a}$	1.6361	0.2835
		$\hat{b}$	1.5225	2.3136
		$\hat{c}$	0.7921	1.0139
		$\hat{k}$	0.0164	0.0161
		$\hat{m}$	2.3961	0.9560
	**|BIAS|**	$\hat{a}$	0.0111	0.1059
		$\hat{b}$	0.1525	1.0201
		$\hat{c}$	0.4153	0.6882
		$\hat{k}$	0.0305	0.0216
		$\hat{m}$	0.0017	0.3487
200	**MSE**	$\hat{a}$	1.3802	0.1665
		$\hat{b}$	2.1323	1.6572
		$\hat{c}$	1.2533	1.0079
		$\hat{k}$	0.0132	0.0120
		$\hat{m}$	1.9351	3.5826
	**|BIAS|**	$\hat{a}$	0.0103	0.0935
		$\hat{b}$	0.1023	0.6272
		$\hat{c}$	0.0012	0.3879
		$\hat{k}$	0.0106	0.0115
		$\hat{m}$	0.0109	0.0103

**Table 2.** The results of simulations conducted for the EOGRNH distribution are reported for τ *=* ( *a = 0.9, b = 1.08*, c = 1.11, l=1.5,m=1.9 ).

n	Est.	Est. Par.	MLE	OLSE
50	**MSE**	$\hat{a}$	0.9266	0.1464
		$\hat{b}$	1.8756	1.1703
		$\hat{c}$	0.8038	0.5601
		$\hat{k}$	0.1281	0.4112
		$\hat{m}$	0.7120	0.2444
	**|BIAS|**	$\hat{a}$	0.3035	0.0697
		$\hat{b}$	0.3494	0.5212
		$\hat{c}$	0.1390	0.2487
		$\hat{k}$	0.0170	0.0946
		$\hat{m}$	0.2799	0.1348
100	**MSE**	$\hat{a}$	0.0996	0.3137
		$\hat{b}$	0.6458	0.3003
		$\hat{c}$	0.4151	0.5734
		$\hat{k}$	0.1137	0.4103
		$\hat{m}$	0.2683	0.2305
	**|BIAS|**	$\hat{a}$	0.0088	0.0516
		$\hat{b}$	0.2739	0.1617
		$\hat{c}$	0.1287	0.2120
		$\hat{k}$	0.0152	0.0835
		$\hat{m}$	0.0911	0.0069
200	**MSE**	$\hat{a}$	0.0607	0.0755
		$\hat{b}$	0.4280	0.2563
		$\hat{c}$	0.2091	0.1364
		$\hat{k}$	0.1025	0.0532
		$\hat{m}$	0.2564	0.1348
	**|BIAS|**	$\hat{a}$	0.0051	0.0303
		$\hat{b}$	0.2469	0.1255
		$\hat{c}$	0.1245	0.1106
		$\hat{k}$	0.0120	0.0086
		$\hat{m}$	0.0046	0.0094

Tables 1 & 2 show the estimates for a range of parameters using (MLE) and (OLS) estimation methods across different sample sizes (50, 100, and 200). The accuracy of the estimates became more evident as the sample size increased. The estimates converge to the true values of the parameters as the sample size increases, reducing the variance of the estimates and enhancing their consistency, as demonstrated by the decreasing mean square error (MSE) values. For the smaller sample (50), the values were less accurate, whereas for the larger samples (100 and 200), the estimates were more reliable, indicating that the MLE and OLSE methods become more accurate and consistent with increasing sample size. This reflects the importance of using larger samples to obtain reliable estimates in statistical analyses.

## 6. Application

In this section we compare our distribution with the Gompertz Nadarajah-Haghighi (GoNH) and Nadarajah-Haghighi (NH) distributions based on a real dataset that records the failure times of 50 components, measured per 1000 hours. ^22^

1.600, 0.058, 2.006, 3.704, 0.078, 0.086, 6.816, 11.020, 0.103, 0.114, 0.148, 7.896, 0.254, 0.381, 0.538, 3.058, 0.590, 3.931, 0.618, 0.645, 0.961, 14.730, 1.228, 2.054, 0.262, 3.076, 0.183,3.147, 3.625, 0.379, 4.073, 0.074, 4.393, 4.893, 0.061, 6.274, 0.102, 0.116, 7.904, 2.804, 10.940, 13.880, 0.570, 15.080, 0.036, 0.574, 9.337, 8.022, 4.534, 0.192.

Goodness-of-fit measures, including the Bayesian information criterion ( **BIC**), Akaike information criterion ( **CAIC**), Cramer Von-Mises (Ⱳ), Anderson Darling ( **Ą**), and Kolmogorov-Smirnov ( **Ҝ-ᵴ**) statistics, are calculated to assess the fit of each model to the data.

Table 3 shows descriptive arithmetic for dataset, while the data in the Tables 4, 5, and 6 demonstrate that the proposed EOGRNH distribution outperforms both the GoNH and NH distributions for the following reasons. The EOGRNH distribution yielded the lowest AIC, BIC, CAIC, and HQIC values, indicating a superior balance between the model complexity and data fit. Additionally, the W value for this distribution is 0.0628, which is lower than the other values, indicating a better fit with the data and stronger support for the hypothesis that the data follows this distribution. The p-values also show that there is no substantial statistical significance, which makes the model better at understanding data. Thus, it is evident that EOGRNH is the most popular of the three distributions examined.

**Table 3.** A descriptive arithmetic for dataset.

Variance	n	Median	Max.	Min.	Mean	Skew.	Kurtosis
***x***	50	1.41	15.08	0.04	3.34	1.37	0.92

**Table 4.** The Ҝ-ᵴ value with its corresponding Ⱳ value and ᵽ -value of dataset.

Distribution	Ⱳ	Ą	Ҝ-ᵴ	ᵽ -value
**EOGRNH**	0.0628	0.4653	0.1010	0.6493
**GoNH**	0.1603	0.9959	0.1438	0.2292
**NH**	0.1764	1.0951	0.1472	0.2068

**Table 5.** MLEs and the values of -2L, AIC, CAIC, BIC and HQIC for dataset.

Distribution	-2L	AIC	CAIC	BIC	HQIC
**EOGRNH**	97.44	204.88	206.24	214.44	208.52
**GoNH**	103.04	214.12	215.01	221.77	217.03
**NH**	103.12	210.24	210.49	214.06	211.69

**Table 6.** MLE parameters with respect to dataset.

Distribution	$\hat{a}$	$\hat{b}$	$\hat{c}$	$\hat{l}$	$\hat{m}$
**EOGRNH**	4.9552	0.0099	0.0940	5.1173	0.3822
**GoNH**	1.3983	0.3844	1.9205	0.2853	-------------
**NH**	3.2474	0.3464	-----------	-----------	-------------

**Figure 5.**
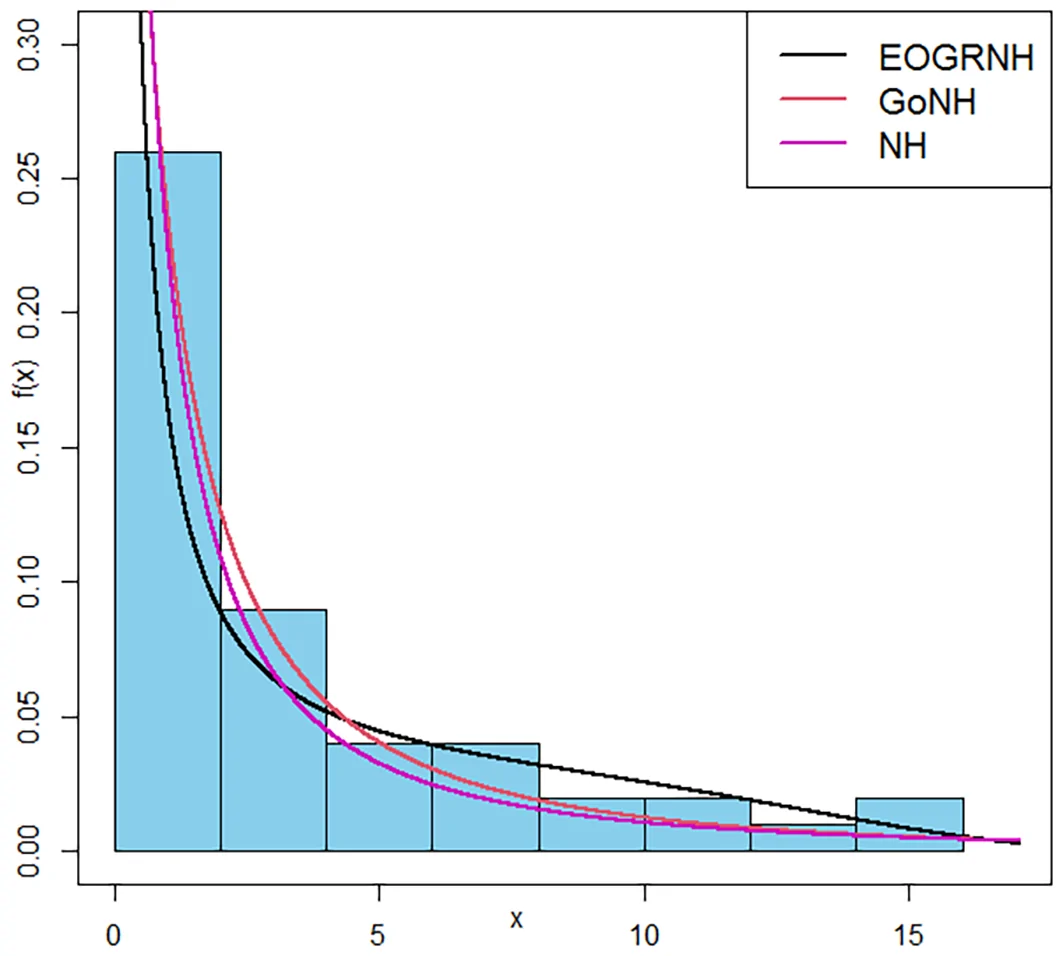
PDF estimated EOGRNH for dataset.

**Figure 6.**
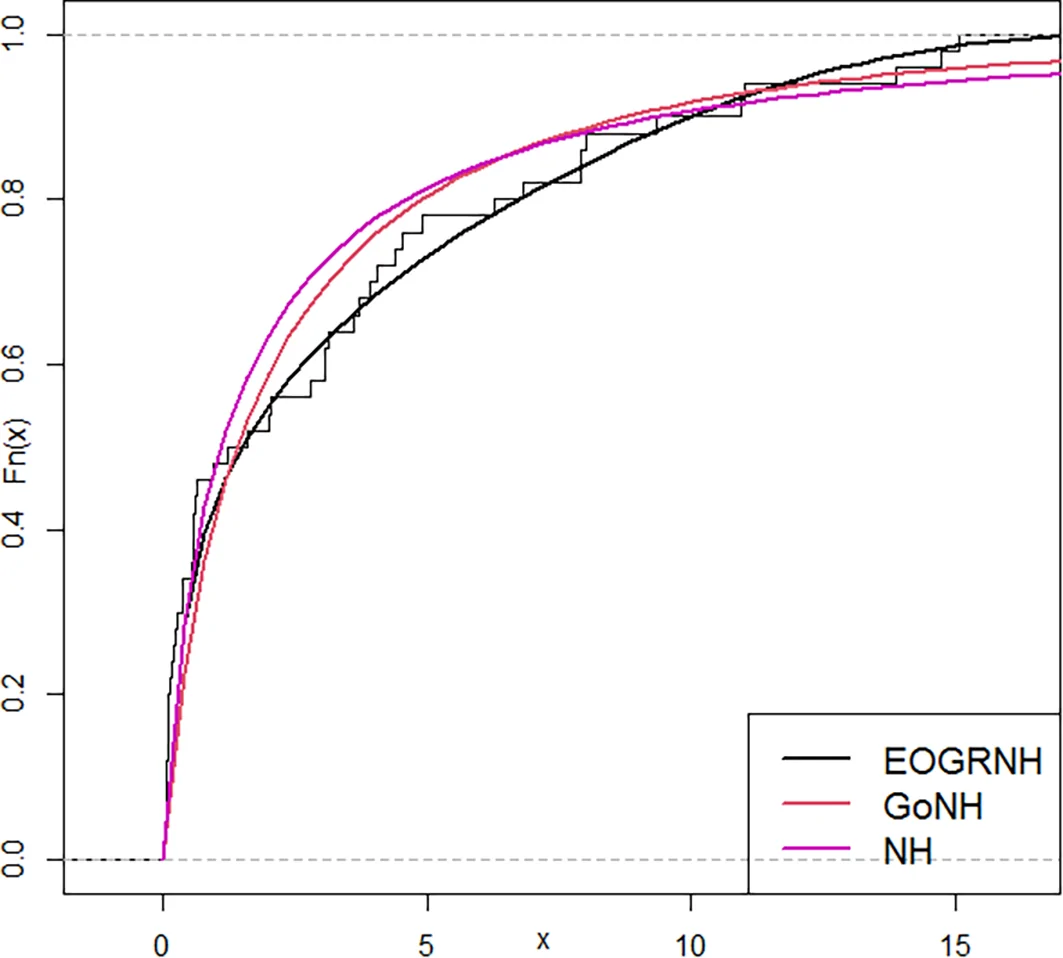
CDF estimated EOGRNH for dataset.

You can look at the attached graphs Figures 5 & 6 to get a better idea of how the data are spread. The EOGRNH curve in Figure 4, which is the distribution density function (PDF), indicates that most values are near zero because there is a lot of data at the bottom and it quickly drops off. The apex of the GoNH curve is less sharp, indicating that the data are more variable. The NH curve looks similar to the GoNH curve, but is not as steep, which means that the values are spread across a wider range. cumulative distribution function (CDF) is presented. The probability increased as the values increased. The EOGRNH, GoNH, and NH curves cluster together slowly, but are formed differently. The EOGRNH curve is convex, therefore, its values cluster quickly at the low end. In contrast, the GoNH and NH curves had gentler slopes, causing their values to spread out more. In general, these graphs show that the EOGRNH, GoNH, and NH models provide information on the nature and spread of the data. This helps you make better choices when performing statistical analysis.

## 7. Conclusion

The EOGRNH distribution outperformed the GoNH and NH distributions in modeling the failure time data, achieving the best fit to thereal data. The maximum likelihood estimator (MLE) is more accurate than ordinary least squares estimation (OLS), particularly when the parameter is large. Because of the superior efficiency and accuracy of the EOGNH distribution, it can be used to analyze reliable data when applying the maximum likelihood estimation (MLE) methodology. In future studies, this work can be built upon by using it to improve statistical inference, or by applying the model to real data.

## Ethics and consent

Since the study did not include sensitive or personal data or human subjects, there is no ethical or approval requirement for this study.

## Data Availability

Zenodo: Real experimental data supporting this study. https://doi.org/10.5281/zenodo.18354028. ^22^ The project contains the following underlying data
• Application of The Failure Time of 50 Components (10^3 hours)-main.zip. (The Failure Time of 50 Components (10^3 hours) Information criteria are tools used to demonstrate a distribution’s flexibility and effectiveness in representing realistic data by comparing it to the performance of other distributions. Some of these criteria, such as the AIC, AIC_c, BIC, and HQIC, will be applied to a data set representing, the data represents the failure time of 50 components (10^3 hours), as it is complete data with a rightward skewed nature). Contains the raw experimental data used to generate Figures (5 and 6) and the results reported in Tables (3, 4, 5, and 6). Zenodo: Simulation and numerical data used in this study. https://doi.org/10.5281/zenodo.18379669 ^23^
1.
Figures (1,2,3,4)_dataset.xlsx (data used to generate Figures 1–4). 2. simulation_data.xlsx (simulation results used in Tables 1 and 2). Data are available under the terms of the Creative Commons Attribution 4.0 International license (CC-BY 4.0).
