# Supplementary material for: New Extension Odd Generalized Rayleigh-Nadarajah Haghighi Distribution with Application and Simulated Data
Source: F1000Res. 2026 Feb 28;15:329. [Version 1] doi: 10.12688/f1000research.173330.1 (PMC13594602; doi:10.12688/f1000research.173330.1)
Supplement: Peer review history

# Reviewer report: Reviewer response for version 1

**DOI:** 10.5256/f1000research.191136.r498930

**Published:** 2026-09-21

**Authors:** Alaa Khlaif Jiheel

**Affiliations:** 1 University of Thi-Qar, Thi-Qar, Iraq

**Overall Assessment**

 The manuscript entitled “New Extension Odd Generalized Rayleigh-Nadarajah Haghighi Distribution with Application and Simulated Data” presents a new probability distribution, referred to as the Extended Odd Generalized Rayleigh-Nadarajah-Haghighi (EOGRNH) distribution, as an extension of the existing Rayleigh-Nadarajah-Haghighi family. The main contribution of the manuscript is the construction of a new flexible probability model intended particularly for modeling lifetime and reliability data. The authors provide a comprehensive theoretical investigation of the proposed distribution, including its probability density and distribution functions, survival and hazard functions, moments, skewness and kurtosis, incomplete moments, order statistics, moment-generating function, quantile function, and Rényi entropy. In addition to the theoretical development, the manuscript addresses the estimation of the unknown parameters of the proposed distribution using Maximum Likelihood Estimation (MLE) and Ordinary Least Squares (OLS) methods. The finite-sample performance of these estimation procedures is investigated through a Monte Carlo simulation study using bias and mean squared error (MSE) as evaluation criteria. The proposed model is also applied to a real dataset consisting of the failure times of 50 mechanical components and is compared with competing probability models, including the Gompertz Nadarajah-Haghighi and Nadarajah-Haghighi distributions.

**Scientific Strengths**

**1**. Development of a new probability distribution: The proposed EOGRNH distribution represents a further extension within the class of flexible lifetime distributions. Its construction provides an additional probability model that may be useful for datasets exhibiting skewness and other non-standard features commonly encountered in reliability and lifetime analysis.

 2. Comprehensive theoretical investigation: The authors derive and investigate a relatively broad range of mathematical and statistical properties of the proposed distribution. This includes its basic reliability functions as well as moments, skewness, kurtosis, incomplete moments, order statistics, the moment-generating function, quantile function, and Rényi entropy.

 3. Parameter estimation: The manuscript considers two estimation approaches, namely MLE and OLS. The inclusion of more than one estimation method provides a useful basis for assessing the behavior of the unknown parameter estimators from different methodological perspectives.

 4. Simulation study: The Monte Carlo simulation provides an empirical assessment of the proposed estimation procedures. The reported results, based on absolute bias and MSE, indicate that the estimation performance generally improves with increasing sample size, supporting the practical behavior of the proposed estimation methods.

 5. Application to real data: The application to failure-time data from 50 mechanical components provides an appropriate practical illustration of the proposed model. The comparison with existing models using several goodness-of-fit and information criteria demonstrates the applicability of the new distribution to reliability data. In the considered dataset, the proposed EOGRNH model reports the smallest AIC, BIC, CAIC, and HQIC values among the competing models.

 6. Reproducibility and supporting data: The availability of the application data together with the simulation and numerical results is a positive feature that enhances the transparency and reproducibility of the study.

**General Comments**

 Overall, the manuscript presents a coherent progression from the construction of the proposed probability distribution to the derivation of its statistical properties, estimation of its unknown parameters, simulation-based assessment, and application to real reliability data. This combination of theoretical and empirical analysis strengthens the statistical contribution of the work. The mathematical development, simulation study, and real-data application provide adequate support for the objectives of the manuscript. I did not identify any substantive methodological issue that would prevent the work from being considered for publication. The few issues that could be addressed concern mainly notation, wording, and presentation and can reasonably be handled during the editorial and final revision stages.

**Recommendation**

 Based on the theoretical development, statistical analysis, simulation study, and real-data application presented in the manuscript, I consider the work to make a useful contribution to the literature on flexible probability distributions and reliability modeling. The manuscript provides a reasonable balance between theoretical development and statistical application, and the proposed distribution is supported by both simulation results and an application to real data. The minor editorial and presentation issues do not substantially affect the scientific contribution or the main conclusions of the study.

 Recommendation: Accept for indexing.

Is the work clearly and accurately presented and does it cite the current literature?

Yes

If applicable, is the statistical analysis and its interpretation appropriate?

Yes

Are all the source data underlying the results available to ensure full reproducibility?

Yes

Is the study design appropriate and is the work technically sound?

Yes

Are the conclusions drawn adequately supported by the results?

Yes

Are sufficient details of methods and analysis provided to allow replication by others?

Yes

Reviewer Expertise:

Statistical Inference, Bayes Estimators, Reliability, Life Testing, and Shrinkage Estimators

I confirm that I have read this submission and believe that I have an appropriate level of expertise to confirm that it is of an acceptable scientific standard.

# Reviewer report: Reviewer response for version 1

**DOI:** 10.5256/f1000research.191136.r463790

**Published:** 2026-09-12

**Authors:** Mundher A Khaleel

**Affiliations:** 1 Tikrit University, Tikrit, Iraq

**Summary and Overall Assessment**

 The manuscript entitled **“New Extension Odd Generalized Rayleigh-Nadarajah Haghighi Distribution with Application and Simulated Data”** introduces a new extension of the Rayleigh-Nadarajah-Haghighi family, referred to as the Extended Odd Generalized Rayleigh-Nadarajah-Haghighi (EOGRNH) distribution. The main objective is to provide a more flexible probability model for lifetime and reliability data.

 The manuscript develops the proposed distribution from both theoretical and applied perspectives. The authors derive several statistical properties, including the distribution and density functions, survival and hazard functions, moments, skewness and kurtosis, incomplete moments, order statistics, moment-generating function, quantile function, and Rényi entropy. Parameter estimation is considered using Maximum Likelihood Estimation (MLE) and Ordinary Least Squares (OLS), and the performance of these estimators is investigated through a Monte Carlo simulation study using bias and mean square error as assessment measures. The proposed model is subsequently applied to failure-time data for 50 mechanical components and compared with the Gompertz Nadarajah-Haghighi and Nadarajah-Haghighi distributions.

 Overall, I found the manuscript relevant and worthwhile, particularly in the context of flexible lifetime distributions and reliability modelling. The study follows a clear general direction from the construction of the proposed model, through its mathematical properties and parameter estimation, to simulation and real-data application. In my opinion, this combination provides a useful assessment of the proposed distribution rather than considering the model from a purely theoretical perspective.

**Strengths of the Manuscript**

**1. Relevant statistical contribution**

 The proposed EOGRNH distribution provides an additional flexible model within the existing class of lifetime distributions. The construction is motivated through the T-X framework and combines the extension of the odd Nadarajah-Haghighi generator with a generalized Rayleigh baseline distribution. This gives the model a potentially useful role in applications involving skewed lifetime and reliability data.

**2. Broad theoretical treatment**

 A strong aspect of the paper is the relatively broad investigation of the mathematical properties of the proposed model. The manuscript considers moments, skewness and kurtosis, the moment-generating function, incomplete moments, quantile function, order statistics and Rényi entropy, in addition to the basic reliability functions. This provides a useful theoretical foundation for the proposed distribution.

**3. Consideration of different estimation methods**

 The use of both MLE and OLS is a positive feature of the study. It allows the parameter estimation problem to be examined from two different perspectives and provides useful information about the finite-sample behavior of the proposed model.

**4. Simulation study**

 The simulation analysis adds practical support to the theoretical development. The authors generate samples from the proposed distribution and evaluate the estimators using absolute bias and MSE over repeated samples. The results generally indicate improved estimation behavior as the sample size increases, which is consistent with the expected behavior of parameter estimation procedures.

**5. Real-data application**

 The application to failure times of 50 mechanical components is appropriate for demonstrating the usefulness of the proposed model in reliability analysis. The authors compare the EOGRNH distribution with both GoNH and NH models using several goodness-of-fit and information criteria. The reported results favor the proposed distribution for this dataset, with the EOGRNH model producing the smallest AIC, BIC, CAIC and HQIC values among the models considered.

**6. Availability of supporting data**

 Another positive aspect is that the authors provide the underlying application data as well as the simulation and numerical data used to produce the reported results. This is valuable for transparency and facilitates further investigation of the proposed model.

**Minor Observations**

 I have only a few minor observations regarding the presentation of the manuscript. Some notation and wording could be polished during the normal editorial preparation of the article, but these points are largely presentational and do not affect the main statistical contribution of the study.

 The mathematical development, simulation analysis, and real-data application provide sufficient support for the objectives of the manuscript. I do not identify a substantive methodological concern that would prevent indexing.

**Overall Evaluation and Recommendation**

 Overall, I consider the manuscript to be a useful contribution to the literature on flexible probability distributions and reliability modelling. The proposed EOGRNH distribution is investigated from several complementary perspectives, and the combination of theoretical properties, parameter estimation, simulation results, and a real-data application strengthens the practical value of the work.

 The manuscript provides a reasonable balance between theoretical development and statistical application. The empirical results also indicate that the proposed model can provide a competitive fit to the reliability data considered in the study.

 The minor observations noted above are mainly editorial in nature and do not, in my opinion, affect the scientific contribution or the main conclusions of the manuscript.

**I therefore recommend approval of the manuscript for indexing.**

**Recommendation: Approved.**

Is the work clearly and accurately presented and does it cite the current literature?

Yes

If applicable, is the statistical analysis and its interpretation appropriate?

Yes

Are all the source data underlying the results available to ensure full reproducibility?

Yes

Is the study design appropriate and is the work technically sound?

Yes

Are the conclusions drawn adequately supported by the results?

Yes

Are sufficient details of methods and analysis provided to allow replication by others?

Yes

Reviewer Expertise:

Mathematical Statistics

I confirm that I have read this submission and believe that I have an appropriate level of expertise to confirm that it is of an acceptable scientific standard.

# Reviewer report: Reviewer response for version 1

**DOI:** 10.5256/f1000research.191136.r482432

**Published:** 2026-05-27

**Authors:** Caner Tanış

**Affiliations:** 1 Necmettin Erbakan University, Konya, Turkey

- The "Background" subsection is written in an overly casual tone and contains typographical errors like "groupd." It should be completely rewritten to be formal, precise, and to clearly state the mathematical contribution of the model.
-  The text suffers from numerous grammatical errors and awkward phrasings throughout. In Section 2, transitioning into unformatted definitions without introductory sentences disrupts the mathematical flow.
- Critical mathematical expressions and expansions on Pages 5 and 6 overlap with the column margins or lack proper line breaks. All equations must be carefully audited, aligned, and confined within standard text margins.
-  In Section 4, the true parameter values used to generate the synthetic data are completely omitted, making the simulation study irreproducible. The authors must declare these baseline setups and include standard criteria like MSEs or Biases.
- Tables 1, 2, and 3 in the application section contain corrupted headings and overlapping, unreadable numeric entries. Essential goodness-of-fit statistics (AIC, BIC, K-S statistic, p-values) must be properly reformatted and presented.
- The authors fail to compare the performance of the EOGRNH model against relevant, close baseline distributions. Fitting a single model to one data set without comparative metrics does not justify a new four-parameter distribution.

- *Is the work clearly and accurately presented and does it cite the current literature?* **No / Partly** (Due to heavy typesetting corruption and a weak literature review).
- *Is the study design appropriate and is the work technically sound?* **Partly** (The theoretical foundation is standard, but the presentation is flawed).
- *Are sufficient details of methods and analysis provided to allow replication by others?* **No** (Due to completely omitted baseline parameter configurations in the simulation study).
- *If applicable, is the statistical analysis and its interpretation appropriate?* **No** (The application tables are corrupted and lack comprehensive comparative metrics).
- *Are all the source data underlying the results available to ensure full reproducibility?* **Partly** (While a data link is provided, the lack of simulation parameters prevents full reproducibility).
- *Are the conclusions drawn adequately supported by the results?* **No** (The lack of a comparative analysis against baseline models fails to justify a new four-parameter distribution).

Is the work clearly and accurately presented and does it cite the current literature?

Partly

If applicable, is the statistical analysis and its interpretation appropriate?

No

Are all the source data underlying the results available to ensure full reproducibility?

Partly

Is the study design appropriate and is the work technically sound?

Partly

Are the conclusions drawn adequately supported by the results?

No

Are sufficient details of methods and analysis provided to allow replication by others?

No

Reviewer Expertise:

Statistics

I confirm that I have read this submission and believe that I have an appropriate level of expertise to confirm that it is of an acceptable scientific standard, however I have significant reservations, as outlined above.
